# Antibody Afucosylation Augments CD16-Mediated Serial Killing and IFNγ Secretion by Human Natural Killer Cells

**DOI:** 10.3389/fimmu.2021.641521

**Published:** 2021-03-16

**Authors:** Alexandros Karampatzakis, Petr Brož, Camille Rey, Björn Önfelt, Gabriela Dos Santos Cruz De Matos, Daniel Rycroft, Ashley Ambrose, Daniel M. Davis

**Affiliations:** ^1^Lydia Becker Institute of Immunology, Faculty of Biology, Medicine and Health, University of Manchester, Manchester, United Kingdom; ^2^Department of Microbiology, Tumor, and Cell Biology, Karolinska Institutet, Stockholm, Sweden; ^3^Science for Life Laboratory, Department of Applied Physics, Kungliga Tekniska Högskolan (KTH) – Royal Institute of Technology, Stockholm, Sweden; ^4^GlaxoSmithKline (GSK), Stevenage, United Kingdom

**Keywords:** Natural Killer cells, antibody dependent cellular cytotoxicity, Fc receptors, afucosylation, cellular activation, immune synapse

## Abstract

One mechanism by which monoclonal antibodies (mAb) help treat cancer or autoimmune disease is through triggering antibody-dependent cellular cytotoxicity (ADCC) *via* CD16 on Natural Killer (NK) cells. Afucosylation is known to increase the affinity of mAbs for CD16 on NK cells and here, we set out to assess how mAb afucosylation affects the dynamics of NK cell interactions, receptor expression and effector functions. An IgG1 version of a clinically important anti-CD20 mAb was compared to its afucosylated counterpart (anti-CD20-AF). Opsonization of CD20-expressing target cells, 721.221 or Daudi, with anti-CD20-AF increased NK cell cytotoxicity and IFNγ secretion, compared to anti-CD20. The afucosylated mAb also caused a more rapid and greater loss of CD16 from NK cell surfaces. Loss of CD16 has recently been shown to be important for NK cell detachment and sequential engagement of multiple target cells. Here, live-cell time-lapse microscopy of individual cell-cell interactions in an aqueous environment and a three-dimensional matrix, revealed that anti-CD20-AF induced more rapid killing of opsonized target cells. In addition, NK cells detached more quickly from target cells opsonized with anti-CD20-AF compared to anti-CD20, which increased engagement of multiple targets and enabled a greater proportion of NK cells to perform serial killing. Inhibition of CD16 shedding with TAPI-0 led to reduced detachment and serial killing. Thus, disassembly of the immune synapse caused by loss of cell surface CD16 is a factor determining the efficiency of ADCC and antibody afucosylation alters the dynamics of intercellular interactions to boost serial killing.

## Introduction

Natural Killer (NK) cells are key players in immune defense against cancerous or virally infected cells. NK cell activation is controlled by the balance of signals from a variety of germline encoded activating and inhibitory receptors (1). Upon activation, NK cells can directly kill diseased cells by secretion of lytic granules that typically contain pore-forming perforin and lytic granzymes (2, 3). NK cells also augment the immune response by secreting immuno-stimulatory cytokines and chemokines including pro-inflammatory interferon gamma (IFNγ) and tumor necrosis factor alpha (TNFα) (4, 5).

Assembly of the immune synapse has been widely studied (6, 7), but how activating signals are terminated and how NK cells dissociate from target cells are understudied elements of the overall process (8). An inability for NK cells to detach from target cells leads to prolonged engagement and increased cytokine production (9). Detachment after lysis allows NK cells to move onto additional target cells (10) and serial killing by NK cells has been revealed by *in-vitro* live microscopy (11, 12). However, while it is clear that NK cell detachment is important for effective NK cell killing, very few specific mechanisms have been described.

Fc receptors allow NK cells to recognize and kill antibody-opsonized target cells independent of other co-stimulatory signals (13); a process called antibody dependent cellular cytotoxicity (ADCC). The ability of NK cells to perform ADCC is critical in targeted immunotherapies based on monoclonal antibodies (mAbs) (14). NK cells respond to immunotherapies through their low affinity immunoglobulin gamma Fc region receptor III (FcγRIII) also known as CD16 (15). Ligand- or cytokine-induced activation triggers rapid and irreversible shedding of CD16 (16–18), which can serve as a regulatory mechanism that inhibits the activation of NK cells and prevents excessive inflammatory responses (19). However, we have recently reported that loss of CD16 from NK cell surfaces can allow NK cells to detach from their targets to enhance serial killing (20).

Cancer cell elimination *via* mAbs involves at least four different mechanisms; ADCC, complement dependent cellular cytotoxicity (CDC), antibody dependent cellular phagocytosis (ADCP) and direct signaling induced cell death (21). ADCC is established to be clinically important and the majority of mAbs approved for treatment in oncology trigger ADCC (22). Specifically, engagement of CD16 has been shown to be important in B-cell chronic lymphocytic leukemia (CLL) and non-Hodgkin's lymphoma, which are treated with anti-CD20 mAbs (23, 24). Most anti-CD20 mAbs used clinically are IgG1 (25, 26), although various Fc modifications such as glycosylation and afucosylation have been tested in attempts to improve immunotherapy efficacy (27). N-glycans that are located on the Fc portion of human IgG are normally highly fucosylated (~90%) (28). ADCC assays have shown that lower Fc fucosylation leads to increased CD16-mediated killing (29, 30). Removing fucose from asparagine 297 of the antibody heavy chain increases the Fc binding affinity to CD16 and improves Fc-triggered effector functions (31, 32). Studies have shown that afucosylated anti-CD20 mAbs triggered more efficient malignant B-cell depletion *in vitro* (33, 34). Afucosylation has also been shown to trigger ADCP of opsonized targets by macrophages at lower mAb concentrations than their unmodified equivalents (35). However, in contrast, Fc afucosylation reduced the binding affinity to complement, and thus CDC associated killing (33, 35). Obinutuzumab (GA101), a highly glycosylated and afucosylated anti-CD20 mAb was developed and shown to have superior cytotoxic activity *in vitro*, and anti-tumor efficacy *in vivo* compared to its native IgG1 form (36). Despite advances in understanding of mAb afucosylation, little is known about how afucosylation affects NK cell contacts with opsonized targets and the killing kinetics of these interactions.

Here, we investigated how afucosylation of an IgG1 anti-CD20 mAb affects primary NK cell lytic responses. We found that an afucosylated anti-CD20 mAb (anti-CD20-AF) induced a more efficient cytolytic response and improved target-specific lysis. In addition, anti-CD20-AF increased CD16 shedding which led to faster killing and increased sequential killing of opsonized targets in comparison to anti-CD20 mAb. We also demonstrated that inhibition of CD16 shedding abrogated the ability of NK cells to kill multiple anti-CD20-AF-opsonized targets. Taken together, enhanced serial killing and efficient target cell elimination indicate that mAb afucosylation can augment NK cell lytic potential and consequently improve the effectiveness of immunotherapies.

## Results

### Afucosylated Anti-CD20 mAb Triggers More Killing and IFNγ Release but Downmodulates CD16

Ligation of the Fc receptor, CD16, on NK cells triggers cytolytic activity and the secretion of pro-inflammatory cytokines (5). Here, we used an afucosylated version of anti-CD20 mAb (anti-CD20-AF) that was afucosylated using *POTTELIGENT* technology (BioWa).

To investigate how afucosylation affected NK cell functional responses, NK cells were incubated with anti-CD20 or anti-CD20-AF mAb opsonized target cells; either the transformed B cell line, 721.221 (hereafter referred to as 221) or the Burkitt's lymphoma, Daudi, cell line (37, 38). It may be important to note that both cell lines tested do not express classical class I MHC proteins and are particularly susceptible to NK cell-mediated lysis. To assess opsonization, 221 cells were incubated with increasing concentrations of each mAb. Binding of the mAbs was detected using a fluorescent secondary antibody with maximal opsonization reached at 10 μg/mL (Supplementary Figure 1A). Opsonization of 221 target cells with anti-CD20 and anti-CD20-AF partially reduced surface CD20 availability to fluorescently labeled anti-CD20 added after opsonization, indicating that loading was equivalent between the two antibodies but that not all surface CD20 was bound by the mAbs when opsonizing targets (Supplementary Figure 1B).

**Figure 1:**
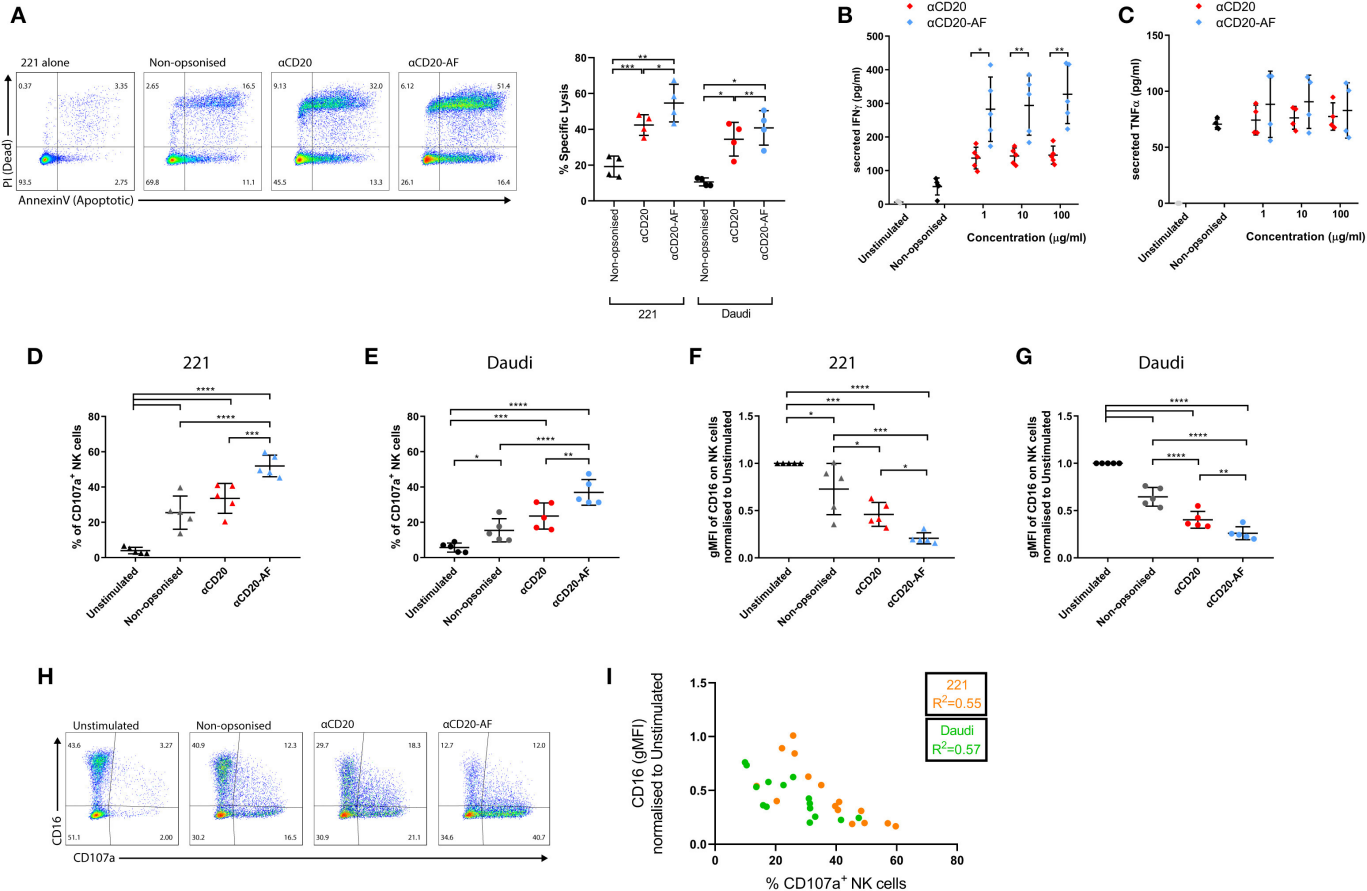
Afucosylated mAb increases NK cell cytotoxicity and IFNγ secretion. NK cells were co-incubated with variably opsonized 221 or Daudi cells for 2 h and then the percentage of specific lysis of targets was assessed. **(A)** Example flow cytometry plots indicate the viability of target cells assessed by Annexin V and PI staining and graph showing the percentage of specific lysis (*n* = 4 distinct donors; mean ± SD). **(B,C)** NK cells were co-incubated with variably opsonized (1, 10, 100 μg/mL) 221 target cells at an Effector to Target ratio (E:T) = 1:3 for 24 h and then the amount of secreted **(B)** IFNγ (*n* = 5 distinct donors; mean ± SD) and **(C)** TNFα (*n* = 4 distinct donors; mean ± SD) were quantified by ELISA. **(D–G)** NK cells were co-incubated with variably opsonized targets at an E:T = 2:1, for 4 h. **(D,E)** The percentage of CD107a^+^ NK cells and **(F,G)** the geometric Mean Fluorescence Intensity (gMFI) of surface CD16 expression, normalized to Unstimulated (US) condition were measured (*n* = 5 distinct donors; mean ± SD). **(H)** Example flow cytometry plots indicating the positive degranulating population (CD107a^+^) and CD16^+^ population in all the conditions. **(I)** Pearson correlation between the percentage of CD107a^+^ NK cells and the gMFI of CD16. *P < 0.05; **P < 0.01; ***P < 0.001, ****P < 0.0001 calculated by one-way ANOVA **(A,D–G)** and multiple *t*-tests ANOVA **(B,C)**.

Human primary NK cells, which had been rested for 6 days after stimulation with low dose IL-2, were co-incubated with opsonized 221 cells at an effector to target ratio (E:T) = 1:3, reflecting immune cells being outnumbered by cancerous cells (39). After 2 h, target cell viability was assessed by staining with Annexin V (apoptotic marker) and propidium iodide (PI), indicative of a permeable membrane (40, 41). The presence of NK cells induced specific lysis of non-opsonized 221 (19.2 ± 5.8%) and Daudi cells (10.5 ± 2.2%; Figure 1A). Opsonization with anti-CD20 mAb increased specific target cell lysis (42.4 ± 5.7% for 221 and 34.4 ± 9.3% for Daudi), whilst anti-CD20-AF opsonized targets were killed more effectively still (54.6 ± 10.5% for 221 and 40.8 ± 9.6% for Daudi; Figure 1A). To assess cytokine secretion, NK cells were co-incubated with 221 target cells opsonized with increasing concentrations of anti-CD20 or anti-CD20-AF mAbs. Non-opsonized target cells caused some secretion of IFNγ (52.7 ± 25.2 pg/mL) whilst 221 cells opsonized with anti-CD20 mAb induced significantly higher secretion of IFNγ (Figure 1B). Opsonization with anti-CD20-AF dramatically increased IFNγ secretion across all concentrations. In contrast, opsonization with either mAb did not trigger any significant level of TNFα secretion (Figure 1C). Thus, opsonization with anti-CD20-AF augments NK cell-mediated specific lysis and secretion of IFNγ relative to anti-CD20.

NK cell cytolytic responses can be assessed by quantification of surface CD107a, a marker of lytic granules, that transiently appears on the surface of degranulating NK cells (42). To assess how afucosylation affects NK cell degranulation, NK cells were co-incubated with target cells and the percentage of CD107a^+^ NK cells was measured. In the absence of target cells, a small fraction of NK cells exhibited some level of staining with an anti-CD107a mAb (4.4 ± 0.8%). However, in the presence of 221 and Daudi target cells 25.5 ± 9.3% and 15.4 ± 6.5% of NK cells degranulated, respectively (Figures 1D,E). Opsonization of 221 cells with anti-CD20 mAb significantly increased the amount of degranulating NK cells (32.4 ± 8.1%), while anti-CD20-AF opsonization further increased degranulating NK cells (50.0 ± 6.8%). Similarly, anti-CD20 opsonized Daudi cells triggered 23.5 ± 7.4% of NK cells to degranulate, while anti-CD20-AF triggered 36.9 ± 7.2% (Figure 1E). Together these data are consistent with the higher affinity anti-CD20-AF mAb triggering more NK cell degranulation.

In parallel, CD16 surface expression was quantified relative to unstimulated NK cells. Co-culture with non-opsonized 221 and Daudi target cells induced significant decreases in CD16 surface expression (Figures 1F,G). This is consistent with previous observations that NK cell activation leads to a loss of CD16 from the cell surface, even in the absence of specific ligation of CD16 (17). Opsonization of 221 target cells with anti-CD20 mAb significantly reduced surface CD16 (54.2 ± 12.7% reduction), whereas targets opsonized with anti-CD20-AF reduced surface CD16 expression to an even greater extent (78.8 ± 5.5% reduction; Figure 1F). Similarly, opsonization of Daudi target cells with anti-CD20 significantly reduced surface expression of CD16 by 59.6 ± 8.8% and anti-CD20-AF reduced this by 73.9 ± 6.8% (Figure 1G). Across both cell lines, degranulation and CD16 surface expression were inversely correlated (221, *R*^2^ = 0.55 and Daudi, *R*^2^ = 0.57; Figures 1H,I) implying that both happen concurrently with NK cell activation. The expression of other NK cell receptors, and integrin LFA-1, were also compared on NK cells co-incubated with Daudi or Daudi cells opsonized with anti-CD20 or anti-CD20-AF. We found that surface expression of the activating receptors NKG2D, NKp30, NKp44, and NKp46, or the integrin LFA-1 were not altered (Supplementary Figure 2). However, NKG2D and NKp30 expression was reduced when NK cells were co-incubated with target cells expressing their respective ligands, Daudi-MICA (43) or K562 (44). Overall, anti-CD20-AF mAb triggers potent NK cell activation beyond that seen with anti-CD20 mAb, resulting in strongly reduced expression of surface CD16 with no effect on other activating receptors.

### Anti-CD20-AF mAb Augments Rapid Serial Killing of Opsonized Targets

**Figure 2:**
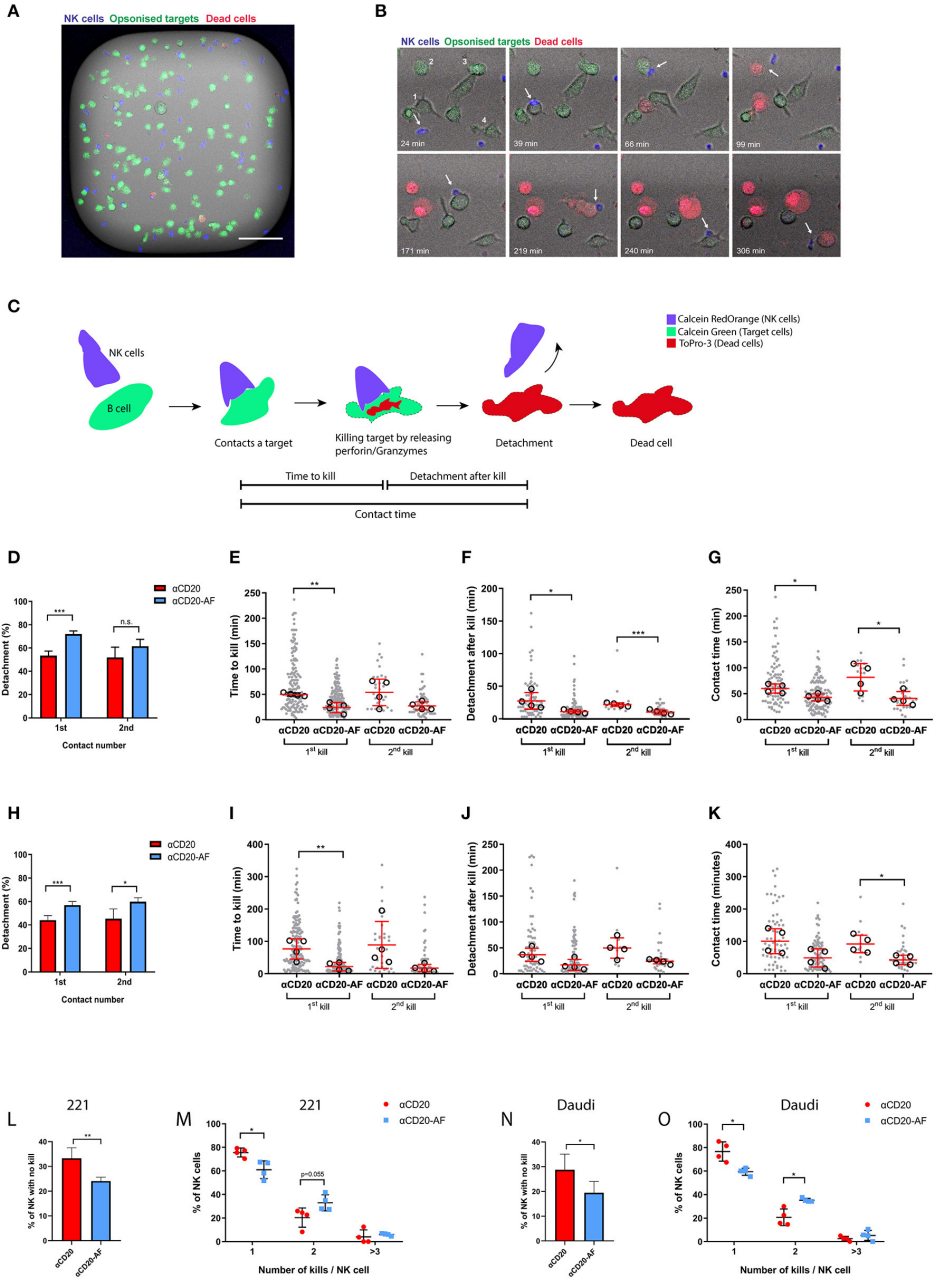
Afucosylated mAb augments serial killing of targets. **(A–O)** NK cells were co-incubated with opsonized 221 or Daudi target cells in microwells at E:T = 1:3 and observed *via* time-lapse microscopy with images captured every 3 min for 6 h. **(A)** Representative image of one microwell (450 × 450 μm) in fluorescence overlaid onto brightfield. NK cells are shown in blue, opsonized target cells in green and dead cells in red (Scale bar, 100 μm). **(B)** Example time course from one enlarged region of a microwell with time indicated (Scale bar, 20 μm). The example sequence shows an NK cell (blue; indicated with an arrow) killing 4 opsonized 221 targets (anti-CD20-AF) sequentially as indicated (1-4). The NK cell encountered the first target (39 min) and the second target (66 min), detached from the second cell after killing (99 min) and encountered a third target (171 min). Lysis of the third target completed after 219 min and then a fourth target was encountered (240 min) that was lysed at 306 min. **(C)** Schematic illustration of an NK cell target interaction that results in a kill with the events observed for time to kill, contact time and detachment time indicated. Analysis of interactions between NK cells and anti-CD20 or anti-CD20-AF opsonized 221 cells **(D–G)** or Daudi cells **(H–K)**. **(D)** Percentage of NK cells which detached from the first and second targets and **(E)** time to kill the first and the second target, **(F)** detachment time after first and second kills of 221 target cells and **(G)** total duration of the first and second contacts. **(H)** Percentage of NK cells which detached from the first and second target and **(I)** time to kill the first and the second Daudi target, **(J)** detachment time after first and second kills of Daudi target cells and **(K)** duration of the first and second contact. **(L)** The percentage of NK cells which did not kill any 221 target cells and **(M)** the percentage of NK cells killing one, two, or ≥ three 221 target cells. **(N)** The percentage of NK cells which did not kill opsonized Daudi targets and **(O)** the percentage of NK cells killing one, two or ≥ three Daudi target cells. (**D,H,L–O**; *n* = 4, distinct donors; Mean ± SD). **(E–G,I–K)** each dot represents an individual cell and each open circle represents the median of different donors (*n* = 4 distinct donors; median ± SD). *P < 0.05; **P < 0.01; ***P < 0.001 calculated by one-way ANOVA **(E–G,I–K)** and *t*-test **(L,N)** and multiple *t*-test **(D,H,M,O)**.

To investigate how mAb afucosylation impacts the killing kinetics of NK cells, we performed live-cell time-lapse microscopy in fibronectin-coated microwells (450 × 450 μm) (45). 221 or Daudi cells were used as target cells and stained with one dye, Calcein Green, before co-incubation with NK cells stained with another dye, Calcein Red-Orange, at an E:T ratio of 1:3. Target cells were also stained with another dye, To-Pro-3, and cell death was indicated by its release (Figures 2A,B). This system enabled detailed quantification of NK cell-target cell interactions, with total contact time, time to kill and time to detachment after kill all assessed (Figure 2C). Example interactions are shown in Figure 2B and Supplementary Videos 1–5. In some cases, target cells were killed very quickly, within 12 min (Supplementary Video 1), while other targets required interactions of almost 3 h to induce lysis (Supplementary Video 2). Detachment is a crucial step in NK cell serial killing but occasionally NK cells can kill a second target without prior detachment from the previous target (Supplementary Video 3). Interestingly, some NK cells killed two targets within a short time span (Supplementary Video 4), and some killed several targets sequentially (Figure 2B and Supplementary Video 5).

Detailed analysis of NK cell-target interactions was performed, using either opsonized 221 or Daudi cells. First contacts between NK cells and anti-CD20 mAb opsonized 221 cells that led to kills resulted in less frequent detachment (53.3 ± 3.9%) than anti-CD20-AF-opsonized 221 cells (71.9 ± 2.6%; Figure 2D), whilst NK cells which contacted and killed a second opsonized target cell were equally likely to detach with either mAb. We then focused on contacts between NK cells and opsonized 221 cells that resulted in kills and examined the duration of lysis and total contact time. Opsonization with anti-CD20-AF significantly reduced the time required to kill target cells, 24 ± 10.2 min, in comparison to anti-CD20, 49.8 ± 3.3 min (Figure 2E). The time taken to kill a second cell was also reduced for 221 targets opsonized with anti-CD20-AF (27.3 ± 7.9 min) compared to anti-CD20 mAb (54 ± 26.1 min). Importantly, detachment time following the first kill was significantly longer with anti-CD20-opsonized 221 cells (28.1 ± 12.8 min compared to anti-CD20-AF, 11.6 ± 2.5 min; Figure 2F). Similarly, detachment after a second kill was faster in the presence of anti-CD20-AF (10.8 ± 3.3 min compared to anti-CD20 mAb, 22.5 ± 2.7 min; Figure 2F). Taken together, both first and subsequent contacts between NK cells and anti-CD20-AF opsonized 221 cells that resulted in a kill were significantly shorter (1st, 42.7 ± 6.6 min and 2nd, 40.8 ± 13.4 min) than with anti-CD20-opsonized 221 cells (1st, 60 ± 8.8 min and 2nd, 81.7 ± 26.4 min; Figure 2G). Altogether, these data indicate that anti-CD20-AF facilitated faster killing and more efficient detachment than anti-CD20 mAb.

To test whether or not these results were generalizable to other target cells, we used Daudi as an alternative CD20-expressing cell. For Daudi, there was a significant difference in the number of cells which could detach after first (56.8 ± 3.1% for anti-CD20-AF compared with 44 ± 3.9% for anti-CD20 mAb) or second contacts (59.8 ± 3.3% for anti-CD20-AF compared with 45.2 ± 8.4% for anti-CD20; Figure 2H). The time taken for NK cells to kill the first target cell was significantly shorter for cells opsonized with anti-CD20-AF (21.7 ± 13.1 min compared to 76.5 ± 31.3 min for anti-CD20 mAb; Figure 2I). While the detachment time was unaffected for first or second kills (Figure 2J), the total duration of the second contact of anti-CD20-AF-opsonized Daudi was significantly shorter (43.1 ± 14.4 min compared to 92.6 ± 27 min for cells opsonized with anti-CD20 mAb; Figure 2K). Collectively, these data indicate that the higher affinity anti-CD20-AF mAb altered the dynamics of intercellular interactions and induced more efficient killing of multiple opsonized target cells.

The total number of target cells killed by an individual NK cell was also quantified. A higher proportion of NK cells did not kill any 221 cells opsonized with anti-CD20 mAb (33.3 ± 4.2%) compared to anti-CD20-AF opsonized targets (24 ± 1.5%; Figure 2L). NK cells incubated with 221 targets opsonized with anti-CD20 mAb predominantly killed a single target (75.5 ± 3.7%). However, more NK cells made sequential kills with 221 target cells opsonized with anti-CD20-AF than with anti-CD20 mAb (32.9 ± 6.7% compared to 20.3 ± 8.1%; Figure 2M). Similarly, more NK cells did not kill any Daudi target cells opsonized with anti-CD20 mAb (28.7 ± 6.3%) compared to anti-CD20-AF (19.4 ± 4.5%; Figure 2N). When NK cell-mediated killing was triggered, anti-CD20 mAb induced mostly single kills (76.7 ± 8.1%) whilst anti-CD20-AF triggered two sequential kills significantly more frequently (35.2 ± 1.5% for anti-CD20-AF compared to 20.7 ± 7% for anti-CD20 mAb) or more than three kills (5.3 ± 4.3%; Figure 2O). Collectively, these data indicate that afucosylated anti-CD20 mAb triggers faster kills, shorter contacts and more efficient detachment which boosts serial killing.

### Anti-CD20-AF mAb Augments Rapid Serial Killing of Opsonized Targets in 3D Environments

**Figure 3:**
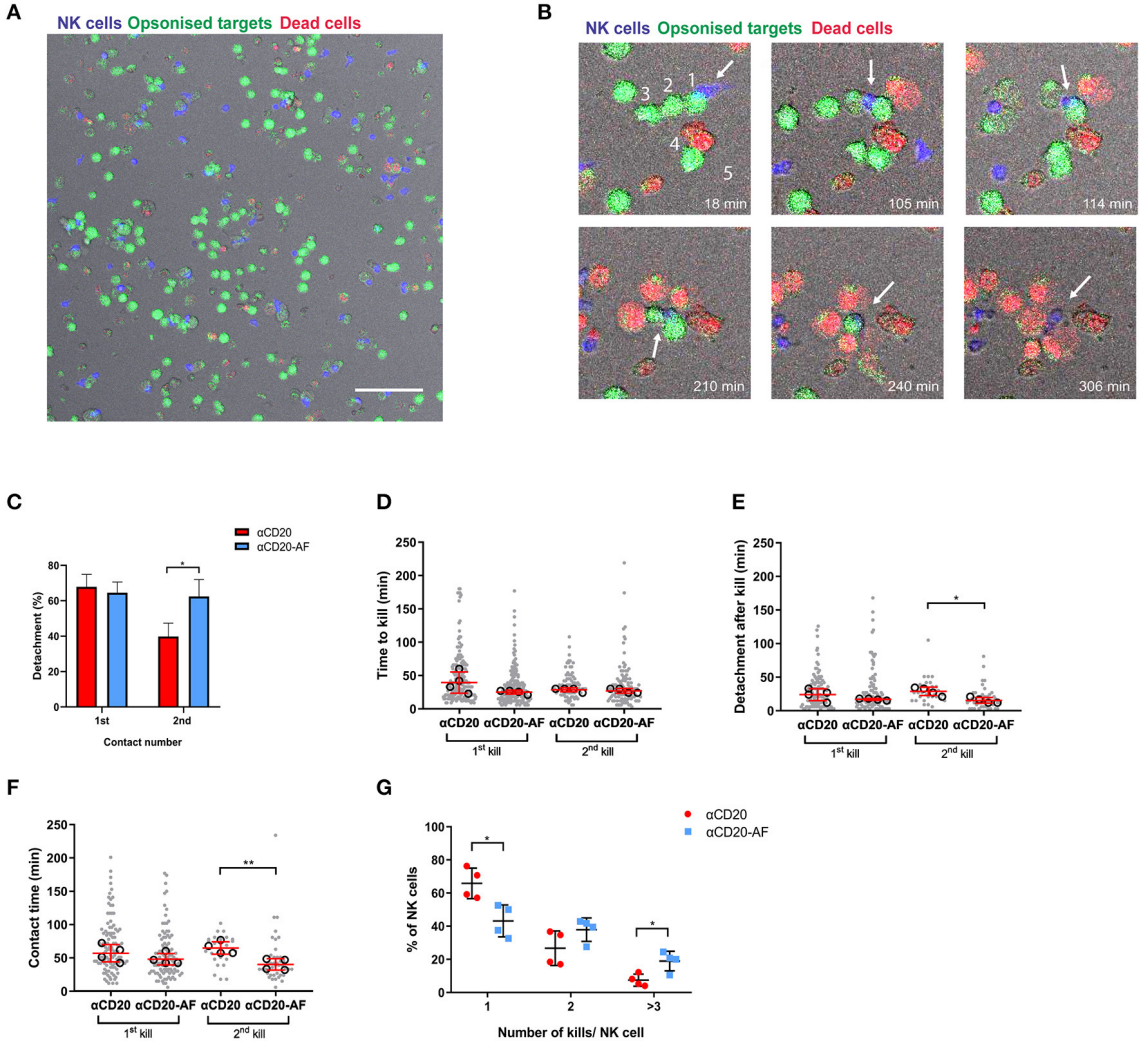
Afucosylated mAb augments serial killing of targets in a 3D environment. **(A–G)** NK cells were co-incubated with opsonized 221 target cells in 3D Matrigel at E:T = 1:3 and observed *via* time-lapse microscopy with images captured every 3 min for 6 h. **(A)** Representative image of 3D Matrigel environment in fluorescence overlaid onto brightfield. NK cells are shown in blue, opsonized target cells in green and dead cells in red (Scale Bar, 100 μm). **(B)** Representative images of enlarged regions from 3D Matrigel, illustrating an NK cell performing serial killing. The example sequence shows how an NK cell (blue; indicated with an arrow) killed 5 targets (coated with anti-CD20-AF) sequentially as indicated with numbers. The NK cell encountered the first target (18 min) and the second target (105 min), detached and encountered a third target (114 min). Lysis of third target occured at 165 min and then the NK cell encountered a fourth target that was lysed at 237 min. A fifth target was encountered at 240 min and lysed at 306 min. **(C)** Percentage of NK cells detaching from the first and second target contacts (mean ± SD), **(D)** time to kill the first and second targets, **(E)** detachment time after first and second kills of target cells and **(F)** the total duration of the first and the second kills. **(D–F)** each dot represents an individual cell and each open circle represents the median of a donor (median ± SD). **(G)** The percentage of NK cells interacting with one, two or ≥ three 221 target cells (mean ± SD). *n* = 4 distinct donors for all. *P < 0.05; **P < 0.01; calculated by ordinary one-way ANOVA **(D–F)** and multiple *t*-tests **(C,G)**.

Intercellular dynamics are known to vary in 3D environments (46). Thus, to analyze NK cell killing kinetics in a more physiological environment, we next sought to investigate whether afucosylation affects NK cell serial killing in a 3D environment. NK cells were labeled with Calcein Red-Orange and co-incubated with 221 target cells stained with Calcein Green opsonized with anti-CD20 mAb or anti-CD20-AF in a 3D Matrigel for 6 h, while dead cells were indicated by To-Pro-3 (Figure 3A). The density of target cells here was higher, compared to microwells tested above, therefore more NK cells were serial killers. In some cases, NK cells killed up to 5 targets sequentially by properly detaching (Figure 3B and Supplementary Video 6), whereas in other cases NK cells killed multiple available neighboring targets without prior detachment (Supplementary Videos 7, 8). Occasionally, NK cells could kill multiple targets within a particularly short time span (Supplementary Video 9).

Interestingly, detachment from first contacts that led to kills was not clearly affected by the mAb used for opsonization but a significantly greater number of NK cells detached from target cells opsonized with anti-CD20-AF and went on to kill a second time (62.4 ± 9.5% compared to 39.8 ± 7.5% for anti-CD20 mAb; Figure 3C). The speed of first kills was slightly, but not significantly faster in the presence of anti-CD20-AF opsonized 221 target cells (25.1 ± 2.8 min compared to 39.3 ± 15.8 min for anti-CD20; Figure 3D). However, there was no difference in the time taken for a second kill. Strikingly, anti-CD20-AF caused significantly faster detachment after second kills (15.3 ± 4.3 min for anti-CD20-AF compared to 28.8 ± 6.1 min for anti-CD20 mAb; Figure 3E). Although the total contact time for first kills was not significantly affected by the afucosylated mAb, second kills of anti-CD20-AF-opsonized target cells were significantly shorter, 39.7 ± 8.7 min, compared to anti-CD20 mAb, 64.5 ± 9.4 min (Figure 3F). Thus, mAb afucosylation boosted NK cell killing kinetics in a 3D microenvironment.

Notably, in a 3D environment, a greater fraction of NK cells engaged in serial interactions with target cells, irrespective of the mAb they were opsonized with. In microwells, 75.5 ± 3.7% and 60.9 ± 7.5% of NK cells were restricted to a single kill for target cells opsonized with anti-CD20 mAb or anti-CD20-AF, respectively. However, this reduced to 65.8 ± 9.1% and 43.1 ± 9.6% in a 3D environment (Figure 3G). In addition, there was a significant increase in the number of NK cells killing three or more target cells opsonized with anti-CD20-AF (18.9 ± 5.9% compared to 7.4 ± 3.6% with anti-CD20; Figure 3G). Collectively, these data establish that the higher affinity anti-CD20-AF mAb triggered more rapid and efficient killing of opsonized targets, and augmented serial killing of multiple targets in microwells and a 3D environment.

### TAPI-0 Inhibitor Triggers IFNγ Secretion, but Blocks Serial Killing

**Figure 4:**
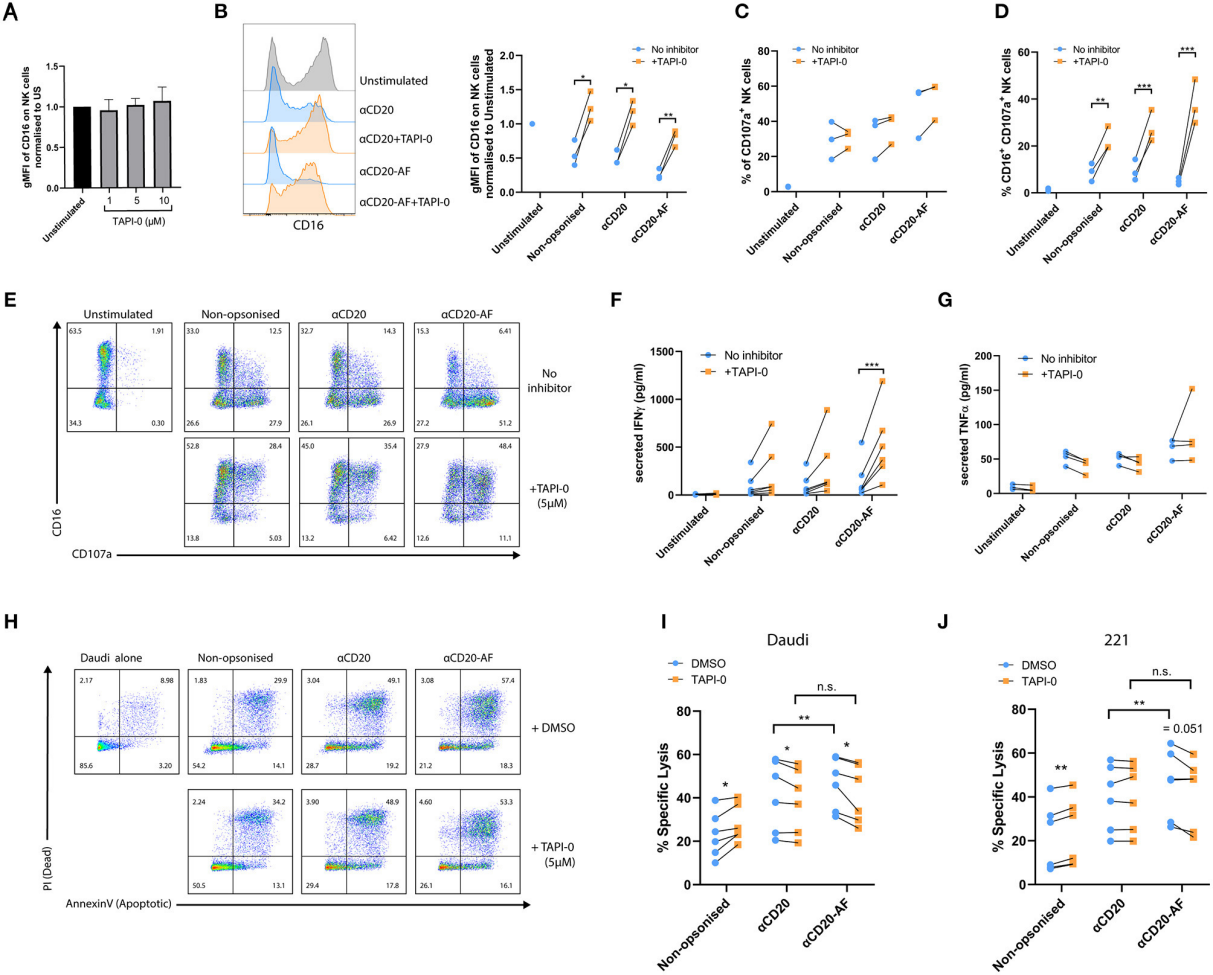
TAPI-0 inhibitor increases the frequency of CD16^+^CD107a^+^ NK cells and IFNγ secretion. **(A–E)** NK cells were co-incubated with opsonized 221 target cells in the presence of the ADAM17 inhibitor, TAPI-0 (5 μM) for 4 h (E:T = 1:2). **(A)** Expression of CD16 on unstimulated resting NK cells (*n* = 4 distinct donors) after 4 h incubation with variable doses of TAPI-0. **(B)** The amount of surface CD16 expression after incubation of NK cells alone or with anti-CD20 or anti-CD20-AF opsonized targets with or without TAPI-0 was assessed by flow cytometry. **(C)** The percentage of CD107a^+^ degranulating and **(D)** CD16^+^CD107a^+^ NK cells (**B–D**; mean ± SD, *n* = 3 distinct donors). **(E)** Example flow cytometry plots showing the percentage of CD16^+^CD107a^+^ NK cells incubated alone or with differently opsonized 221 target cells in the absence or presence of TAPI-0 inhibitor. **(F,G)** NK cells were co-incubated with opsonized 221 target cells in the absence or presence of TAPI-0 inhibitor for 24 h and the amount of **(F)** IFNγ (mean ± SD, *n* = 4 distinct donors) or **(G)** TNFα secreted, assessed by ELISA (mean ± SD, *n* = 4 distinct donors). **(H–J)** NK cells were co-cultured with target cells (Daudi or 221) in the presence of DMSO or TAPI-0 inhibitor at E:T = 1:3 for 2 h. **(H)** Example flow cytometry plots indicating the viability of Daudi target cells assessed by Annexin V and PI staining. Graphs show percent of specific **(I)** Daudi and **(J)** 221 target lysis measured by double staining of Annexin V and PI. *n* = 6 distinct donors. *P < 0.05; **P < 0.01; ***P < 0.001, calculated by multiple *t*-tests **(B–D,F,G,I,J)**.

Shedding of CD16 on activated NK cells *via* the metalloproteinase ADAM17 has been described to aid serial engagement of multiple opsonized targets (20). Using TAPI-0, we investigated how inhibition of CD16 shedding affected NK cell function. NK cells were incubated alone or with 221 targets or 221 targets opsonized with anti-CD20 or anti-CD20-AF for 5 h in the presence of TAPI-0 inhibitor after which we measured surface expression of CD16 and the marker of NK cell degranulation, CD107a. While CD16 expression was not affected on unstimulated NK cells with varying doses of TAPI-0 (Figure 4A), CD16 shedding following co-incubation with 221 cells was inhibited in all conditions in the presence of TAPI-0 (5 μM). TAPI-0 completely inhibited CD16 shedding and even resulted in increased CD16 expression on NK cells co-incubated with non-opsonized or anti-CD20 opsonized 221 cells. However, CD16 levels were still reduced by 20.2 ± 12% in the presence of anti-CD20-AF opsonized 221 cells despite inhibition with TAPI-0 (Figure 4B and Supplementary Figure 3). TAPI-0 did not affect the fraction of NK cells expressing CD107a, indicating that degranulation was unaffected (Figure 4C and Supplementary Figure 3). Accordingly, TAPI-0 caused a significant increase in the number of CD107a^+^CD16^+^ NK cells, indicating that degranulating NK cells retained surface CD16 (Figures 4D,E). In addition, TAPI-0 increased the secretion of IFNγ across all conditions, although due to donor variability this difference was not statistically significant except for anti-CD20-AF opsonized target cells, where the presence of TAPI-0 inhibitor increased IFNγ secretion from 161 ± 199 to 524 ± 378 pg/mL (Figure 4F and Supplementary Figure 3). In contrast, TAPI-0 did not have any significant effect on the secretion of TNFα (Figure 4G). Thus, inhibition of CD16 shedding with TAPI-0 sustained expression of CD16 and boosted IFNγ secretion especially in the presence of 221 target cells opsonized with anti-CD20-AF.

To investigate whether inhibition of CD16 shedding impacts NK cell-mediated lysis, TAPI-0 inhibitor was added to NK cells co-incubated with target cells (E:T = 1:3) for 2 h. In the presence of non-opsonized Daudi and 221 cells, specific lysis was slightly increased by TAPI-0 for all donors (221; 23.0 ± 10.4% vs. 28.0 ± 8.6% and Daudi; 21.2 ± 15.4% vs. 23.7 ± 15.5%). TAPI-0 had no effect on specific lysis of anti-CD20-opsonized 221 target cells, but caused a slight but significant reduction in lysis of Daudi cells (221; 41.1 ± 16.3% vs. 38.9 ± 14.9% and Daudi; 39.8 ± 15.1% vs. 40.0 ± 15.1%). Interestingly, TAPI-0 inhibitor caused a small and significant reduction in the killing of Daudi and 221 cells opsonized with anti-CD20-AF (221; 46.6 ± 12.0% vs. 41.6 ± 13.4% and Daudi; 45.7 ± 15.6% vs. 42.2 ± 15.6%; Figures 4H–J). As before, there was a significant increase in specific lysis of both target cell lines when opsonized with anti-CD20-AF, compared with anti-CD20. However, this difference was eliminated in the presence of TAPI-0 suggesting that CD16 cleavage plays a role in the increased cytotoxicity observed with the afucosylated mAb. These changes in cytotoxicity are small, but measuring the effects of serial killing in bulk assays is challenging, so we next assessed the impact of TAPI-0 on the killing of anti-CD20-AF opsonized targets using time-lapse microscopy.

**Figure 5:**
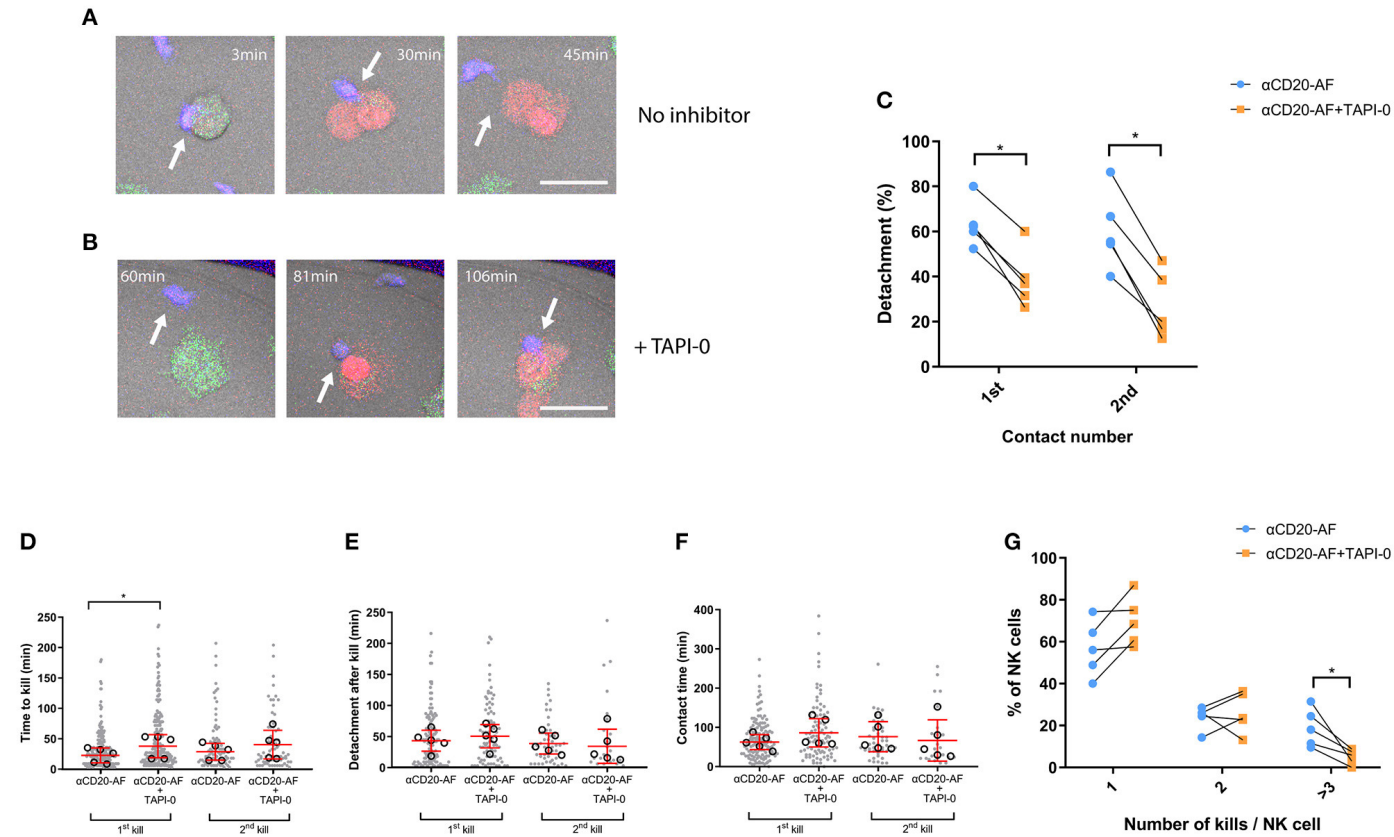
TAPI-0 inhibitor blocks efficient detachment and efficient serial killing. **(A–G)** Primary NK cells were co-incubated with anti-CD20-AF-opsonized 221 target cells in microwells at E:T = 1:3 with or without TAPI-0. Images were acquired by confocal microscopy and captured every 3 min for 8 h. **(A,B)** Example sequence illustrates how TAPI-0 blocks efficient detachment. **(A)** Stained primary NK cell (blue; indicated with an arrow) engaging with a 221 target cell opsonized with anti-CD20-AF in the absence of TAPI-0 inhibitor. NK cell quickly lyses the target cell at 30 min and detaches at 45 min (Scale bar, 5 μm). **(B)** Stained primary NK cell engaging with a 221 target cell opsonized with anti-CD20-AF at 81 min in the presence of TAPI-0 (5 μM), the contact is prolonged until at least 106 min (Scale bar, 5 μm). Graph shows **(C)** the percentage of detachment from the first and second target cells contacted (Mean ± SD, *n* = 5 distinct donors). Graphs show **(D)** the time to kill the first and the second 221 target, **(E)** detachment time after first and second kills of 221 target cells and **(F)** total duration of the first and second kill contacts (each dot represents an individual cell and each open circle represents the median from a single donor, median ± SD, *n* = 5 distinct donors). Graph shows **(G)** the percentage of NK cells interacting with one, two or ≥ three 221 target cells in the presence or absence of TAPI-0. (Mean ± SD, *n* = 5) *P < 0.05 calculated by ordinary one-way ANOVA **(D)** and multiple *t*-tests **(C,G)**.

Shedding of CD16 has been shown to be one factor which can control the disassembly of immune synapses (20), and we next investigated the effect of TAPI-0 on the dynamics of interactions between NK cells and opsonized 221 target cells. As previously, NK cells and 221 target cells were stained with differently colored Calcein dyes, co-incubated in fibronectin-coated microwells, and imaged by time-lapse microscopy (Figures 5A,B). Cells were either left alone or treated during acquisition with 5 μM TAPI-0 inhibitor to block CD16 shedding. TAPI-0 dramatically reduced the percentage of NK cells which detached following both the first (63.4 ± 10% without TAPI-0 and 38.8 ± 12.8% with TAPI-0) and second contacts (60.6 ± 17.2% without TAPI-0 and 26.9 ± 15% with TAPI-0; Figure 5C) that resulted in kills of 221 cells opsonized with anti-CD20-AF. It is possible that sustained interaction with target cells in the presence of TAPI-0 is directly responsible for increasing IFNγ secretion, although this is hard to test directly. An example of TAPI-0 preventing an NK cell from detaching is shown in Supplementary Video 10. In the absence of TAPI-0, NK cells normally detach as shown in Supplementary Video 11. Of the contacts which resulted in kills and detachment, the presence of TAPI-0 did significantly impact the time for the first kill but not significantly for the second kill (Figure 5D), the time taken to detach after killing (Figure 5E), nor the total time of contact (Figure 5F). It's important to emphasize that the data shown in Figures 5E,F only represents interactions where cells detached. As shown by Figure 5C, most cells remained attached in the presence of TAPI-0 and therefore the overall times for cells to detach after killing and the total contact times are understated by the values shown. Accordingly, the number of NK cells able to kill three or more anti-CD20-AF opsonized target cells (18.9 ± 9.1%) was dramatically reduced in the presence of TAPI-0 (5 ± 3.5%; Figure 5G and Supplementary Video 12). Thus, inhibition of CD16 shedding by TAPI-0 causes less efficient NK cell detachment inhibits serial killing of target cells opsonized with anti-CD20-AF.

## Discussion

ADCC by NK cells is a significant contributor to both clinical and *in vivo* anti-tumor activity of anti-CD20 mAbs (47). The Fc portion of mAbs is responsible for ligation to the activating Fc receptor CD16 on NK cells, and therefore dictates their ADCC response. Here, we investigated how afucosylation of an anti-CD20 mAb induced potent NK cell activation resulting in more efficient target cell killing and IFNγ secretion. Critically, we observed an increase in the serial killing of multiple NK cell susceptible targets with an afucosylated mAb, anti-CD20-AF. Anti-CD20-AF triggered strong shedding of surface CD16, increasing the fraction of NK cells able to detach effectively from target cells and engage in sequential killing.

Clinically, humanized IgG1 mAbs that have moderate affinity with CD16 are predominantly used (48). However, by Fc engineering of mAbs (49, 50), many variants of anti-CD20 mAbs have been produced and tested (51). Here we compared an afucosylated version of anti-CD20 with its native form. We found that afucosylation led to more efficient elimination of opsonized B cells compared to the native anti-CD20 mAb. This is consistent with previous work showing that afucosylated Obinutuzumab increased specific lysis of opsonized neoplastic B cells (36). Stimulation with anti-CD20-AF also induced greater NK cell degranulation, confirming a previous study where afucosylated mAb opsonized Raji B cells induced strong degranulation (52). This increased degranulation has been shown to be a result of increased downstream phosphorylation (34). Moreover, our results agree with findings that showed afucosylated Obinutuzumab induced superior IFNγ secretion (52). In contrast, anti-CD20-AF did not induce more TNFα secretion compared to the native antibody. Overall, this implies that an afucosylated version of anti-CD20 mAbs can be beneficial clinically.

It has been established that surface CD16 is downregulated on activated NK cells. CD16 is shed after cleavage by ADAM17 (17, 18) or MMP25 (16). Indeed, here, anti-CD20-AF triggered greater reduction of CD16 surface expression on activated NK cells compared to the native anti-CD20 mAb. Shedding of CD16 has been suggested to function as a regulatory mechanism of activated NK cells (53). However, we recently described that CD16 shedding can boost serial killing (20) and in this study, we found that effective serial killing still occurred alongside significant downregulation of CD16 expression. Previously, Srpan et al. found that even a 60% downregulation of surface CD16 did not prevent NK cells secreting cytotoxic perforin in response to further CD16 stimulations (20). Here, we observe even greater shedding of CD16 following NK cell stimulation with high-affinity anti-CD20-AF but still see effective serial killing. Whether or not further reduction in CD16 expression during subsequent interactions can be detrimental to prolonged serial killing is unclear. Altogether, it seems likely that effective long-term serial killing would involve stimulation through more than one NK cell activating receptor.

Inhibition of ADAM17 with TAPI-0 could not completely restore surface CD16 on activated NK cells in the presence of anti-CD20-AF opsonized target cells. This higher affinity mAb may trigger loss of surface CD16 through an additional mechanism alongside shedding, which would not be prevented by TAPI-0, such as endocytosis. Indeed, strong stimulation with an anti-CD16 mAb (clone; 3G8) has been shown to lead to CD16 endocytosis (18) and co-incubation with Raji cells opsonized with the afucosylated mAb, Obinutuzumab, resulted in targeted lysosomal degradation of CD16-associated signaling elements (52). Therefore, higher affinity anti-CD20-AF may trigger even greater reductions of CD16 than the native antibody by employing additional mechanisms to reduce CD16 surface expression and enhance NK cell killing kinetics.

NK cells are well-established as being able to eliminate multiple targets sequentially. Here, we found that anti-CD20-AF facilitated faster killing and shorter contacts with opsonized targets as well as efficient serial killing. Previously, an anti-CD33 leukemic mAb was Fc engineered to increase affinity to CD16 using a triple mutation Ser293Asp/Ala330Leu/Ile332Glu that resulted in improved killing speed and boosted serial killing (54). Another study used a microscopy-based cytotoxicity assay to observe that the afucosylated mAb Obinutuzumab, increased the number of NK cell serial killing events of a CD20-expressing cell line (WIL-2S) alongside a decreased target cell lysis time (34). When we used a more physiologically relevant 3D model system, anti-CD20-AF caused a significant increase in multiple serial kills compared to the native anti-CD20 mAb. Collectively, these data support that Fc afucosylation boosted the ability of NK cells to rapidly engage and kill several opsonized target cells sequentially.

Serial killing of multiple target cells partially relies on efficient detachment (20). Inhibition of CD16 shedding with TAPI-0 had a dramatic effect on the ability of NK cells to detach from anti-CD20-AF opsonized target cells. In turn, efficient detachment boosted more efficient serial killing. Preventing CD16 shedding by ADAM17 inhibition with either anti-ADMA17 mAb (MEDI3622) or specific inhibitor (BMS566394) also elevated the amount of secreted IFNγ, which could lead to excessive immune activation (17, 55). CD16 shedding may, in some cases, not be essential for NK cell detachment and serial killing of targets. Indeed, Zhu et al. demonstrated that non-cleavable CD16 did not affect NK cell detachment from adherent epithelial cells *in vitro* (56).

Anecdotal observations of cellular interactions in the presence of afucosylated anti-CD20 mAb raised several interesting questions. For example, one question is whether or not a new available target cell in close proximity augments NK cell detachment and serial engagement (Supplementary Videos 1, 6–9, 12) (12), perhaps by triggering the relocation of activating receptors or by generating forces to move away. In addition, some NK cells quickly kill the first target cell and then more slowly kill a second target cell which raises the question as to whether or not the mechanisms of killing can vary from one interaction to another. This would be consistent with previous work showing that an initial kill may occur through Granzyme B release, while further lytic interactions may happen *via* receptor-mediated killing (57).

In summary, anti-CD20-AF mAb increased the ability of NK cell cells to lyse opsonized targets in comparison to anti-CD20. Crucially, anti-CD20-AF induced strong CD16 downregulation which allowed NK cells to detach more efficiently following a kill and thus altered the dynamics of interactions to enhance the sequential engagement of target cells.

## Materials and Methods

### Human Primary NK Cells

Primary human NK cells were isolated from the peripheral blood of healthy donors from the National Blood Service under ethics license REC 05/Q0401/108 (University of Manchester, Manchester, UK). In brief, peripheral blood monocyte cells (PBMC) were purified by density gradient centrifugation using Ficoll-Paque (GE Healthcare, Life Sciences). Primary human NK cells were subsequently isolated from PBMCs by negative magnetic selection, using the human NK cell isolation kit (Miltenyi Biotec). Isolated cells were cultured at 37°C and 5% CO_2_ at 10^6^ cells/mL in clone media [Dulbecco's Modified Eagle Medium (DMEM) medium containing 30% Ham's F12 nutrient mixture, 10% human serum, 1% Non-essential amino acids, 1 mM sodium pyruvate (all Sigma-Aldrich), 2 mM L-glutamine, 50 U/mL penicillin/streptomycin, 50 μM 2-mercaptoethanol (all Gibco)]. Primary NK cells were also stimulated with 200 U/mL IL-2 (Roche) and rested for 6 days prior to experiments. NK cells rested in low-dose IL-2 had comparable CD16 expression to freshly isolated NK cells (Supplementary Figure 4).

### Cell Line Culture

Daudi (Burkitt's lymphoma) and 721.221 (B lymphoblastoid) cell lines were used as NK cell susceptible targets and purchased from ATCC. Cells were cultured at 37°C and 5% CO_2_ in media comprising RPMI 1640 medium supplemented with 10% FBS, 1% Non-essential amino acids, 1 mM sodium pyruvate (1%), 2 mM L-glutamine (1%) and 50 U/mL penicillin/streptomycin (1%). All cell lines were routinely tested for mycoplasma infection using a PCR-based kit (PromoCell) that utilizes specific primers designed from DNA sequences coding for highly conserved ribosomal RNAs (16S-rRNA). All cells tested were negative.

### Monoclonal Antibody Production and Target Opsonization

Human IgG1 anti-CD20 monoclonal antibodies were generated based on publicly available sequences (Drugbank Accession: DB00073). Anti-CD20 was produced using HEK293 6E cells and anti-CD20-AF was produced with *POTELLIGENT* Technology licensed from BioWa, Inc. This technology involves the reduction of the amount of fucose in the carbohydrate structure of an antibody using a proprietary fucosyl transferase-knockout CHO cell line as a production cell. Evidence shows that this technology can enhance ADCC activity of an antibody *in vitro* (58, 59). Opsonization was based on incubation of target cells with mAbs for 45 min at 37°C. To assess target cell opsonization, cells were washed with 1% FBS/PBS, fixed with 4% PFA at room temperature for 10 min and blocked with 2% human serum/PBS (Sigma-Aldrich) for 10 min at 4°C. Following target cell opsonization mAbs were labeled with Alexa Fluor 488-conjugated to goat anti-human IgG F(ab')2 fragment specific (Jackson ImmunoResearch) or a second anti-CD20 mAb was added (anti-CD20 PE; clone 2H7, Biolegend) for 20 min at 4°C. Unless indicated otherwise, all isotype mAbs were used at 10 μg/mL based on experiments that defined the saturating concertation of mAb target opsonization.

### Cytokine Detection by Enzyme-Linked Immunosorbent Assay

Primary NK cells were co-incubated with opsonized target cells, and TAPI-0 (5 μM) where indicated, in polystyrene flat bottom 48-well plates (Nunc) for 24 h at 37°C. Where indicated, TAPI-0 in DMSO (stock solution concentration 10 mg/mL) was added such that the final concentration of the inhibitor was 5 μM. Cell supernatants were collected and centrifuged at 500 × g for 5 min at 4°C to remove cell debris. Secretion of IFNγ and TNFα were quantified in the supernatants using ELISA (DuoSet, R&D Systems) according to manufacturer's instructions.

### Annexin V/Propidium Iodide Staining for Dead Cells

In each co-culture, NK cells were labeled with cell proliferation dye (Cell Trace Violet, CTV, Thermo Fisher). Briefly, cells were washed three times in 5 mL of cold RPMI and stained with 5 μM Cell Trace Violet dye in warm RPMI for 15 min at 37°C (E:T = 1 (10^5^):3 (3 × 10^5^). NK cells were co-incubated with opsonized 221 and Daudi cells at E:T ratio 1:3 in a 48-well plate for 2 h at 37°C with TAPI-0 added where indicated. The cells were aspirated and washed once with cold Annexin V binding buffer (25 mM CaCl_2_, 1.4 M NaCl, 0.1 M HEPES). Each condition was then stained with 2 μl of Annexin V-APC (BioLegend), incubated for 15 min at room temperature, and washed with Annexin V binding buffer. 20 μl of PI (1 μg/mL) was then added to each tube and samples were immediately analyzed with BD FACS LSRFortessa™ X-20 flow cytometer (BD Biosciences) and analyzed by FlowJo V10 software (BD).

### Flow Cytometry

To assess degranulation, NK cells were co-incubated with opsonized target cell lines in the presence of GolgiPlug (1/1000 dilution, BD Biosciences), monensin (1/1000 dilution, Biolegend) and anti-CD107a PE mAb (clone H4A3, BD biosciences) or an isotype-matched control (PE-IgG1κ; clone MOPC-21, BD) for 4 h at 37°C. After incubation, cells were washed and stained with Zombie NIR viability dye (Biolegend), anti-CD56-AF488 mAb (clone HCD56, Biolegend), anti-CD107a-PE (clone H4A3) and anti-CD16-AF647 (clone; B.731, Biolegend) (42). To assess expression of other proteins, NK cells were co-incubated with opsonized target cell lines for 4 h, then washed and stained with Zombie NIR viability dye (Biolegend) and LFA-1-PE (Clone M24), NKp46-PercP-Cy5.5 (Clone 9E2) and NKp44-APC (Clone P44-8) or NKp30-AF647 (Clone P30-15) and NKG2D-PE (Clone 1D11, all from Biolegend). Isotype-matched control mAbs were also used accordingly (mouse IgG1 isotype control; clone MOPC-21, Biolegend). Finally, cells were fixed in 2% PFA/PBS and staining was assessed using a BD FACS LSRFortessa™ X-20 flow cytometer and analyzed by FlowJo V10 software (BD Biosciences). The gating strategy used for all flow cytometry assays is shown in Supplementary Figure 5.

### Live Time-Lapse Imaging in Microwells or in 3D Matrigel

Sterile microwells were coated with 10 μg/mL fibronectin (Sigma). NK cells were stained with 1 μM Calcein Red-Orange (Invitrogen) and opsonized targets were stained with 1 μM Calcein Green (Invitrogen). To stain with calcein dyes, cells were first washed three times with 10 mL RPMI and then incubated with the dye solution (1 μM) in RPMI for 15 min at 37°C. Labeled NK cells were then added to opsonized target cells at E:T = 1:3 in clone media supplemented with 1 μl To-Pro-3 per well (Thermo Fisher) to discriminate dead cells. Where indicated, 5 μM TAPI-0 (Santa Cruz Biotechnology) was added just before acquisition, the absence of the inhibitor was used as a control for Figure 4, while a vehicle only, DMSO, control was used in Supplementary Figure 3. For imaging in microwells (450 × 450 × 300 μm), stained cells were mixed and immediately added. For imaging in 3D Matrigel (Corning Matrigel Matrix), cells were resuspended in a cold Matrigel solution, diluted 1:4 with clone media and added to 8-well chamber slides (1.5 Lab-Tek II; Nunc). Time-lapse imaging was performed at 37°C and 5% CO_2_ for 6 or 8 h with an image acquired every 3 min. Imaging was performed using a Leica TCS SP8 inverted confocal microscope with a 20x/0.75NA objective (SP8, Leica Biosystems) using a white light laser. Fluorescent and bright-field images were merged and cell-cell interactions were analyzed manually using ImageJ. Microwells were produced in-house and used as previously described (10).

### Statistical Analysis

For each dataset, a D'Agostino and Pearson omnibus test or Shapiro-Wilk normality test was used to evaluate whether the obtained values were normally distributed. The statistical significance between two groups of data with normal distribution was examined using a two-tailed Student's *t*-test. The statistical significance between three or more conditions was assessed by one-way ANOVA. Differences were defined as non-significant where *P* ≥ 0.05 and were statistically significant where ^*^P < 0.05; ^**^P < 0.01; ^***^P < 0.001; and ^****^P < 0.0001. All statistical analyses were performed using Prism v8.0 (GraphPad).

## Data Availability Statement

The original contributions generated in the study are included in the article/Supplementary Material, further inquiries can be directed to the corresponding author/s.

## Ethics Statement

The studies involving human participants were reviewed and approved by REC 05/Q0401/108. The patients/participants provided their written informed consent to participate in this study.

## Author Contributions

AK, PB, CR, and AA: acquisition, analysis, and interpretation of data. AK, DR, AA, and DD: wrote the manuscript. All authors: discussed, reviewed and edited the manuscript, contributed to the concept of this work, and approved the submitted version.

## Conflict of Interest

DC and DR were employed by GlaxoSmithKline. The remaining authors declare that the research was conducted in the absence of any commercial or financial relationships that could be construed as a potential conflict of interest.
