# Supplementary material for: Antibody Afucosylation Augments CD16-Mediated Serial Killing and IFNγ Secretion by Human Natural Killer Cells
Source: Front Immunol. 2021 Mar 16;12:641521. doi: 10.3389/fimmu.2021.641521 (PMC8008054; doi:10.3389/fimmu.2021.641521)
Supplement: Supplementary file 13 [file Data_Sheet_1.docx]

Supplementary Material

##
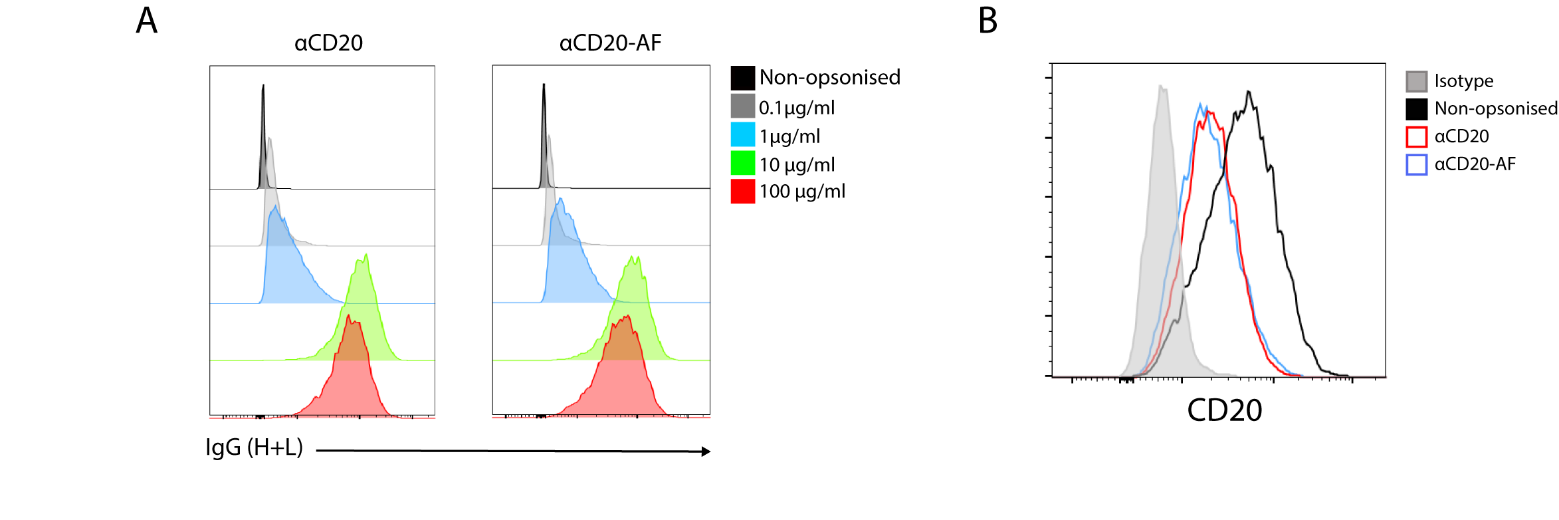
Supplementary Figures

**Supplemental Figure 1. (Α)** Opsonisation of 221 target cells with increasing concentrations of activating mAbs, anti-CD20 and anti-CD20-AF. Histograms show the level of target cell opsonisation in the presence of increasing concentration of each activating mAb. **(B)** Histograms show the CD20 expression on non-opsonised 221 target cells compared to opsonised with activating mAbs, where CD20 is masked by activating mAbs.

**
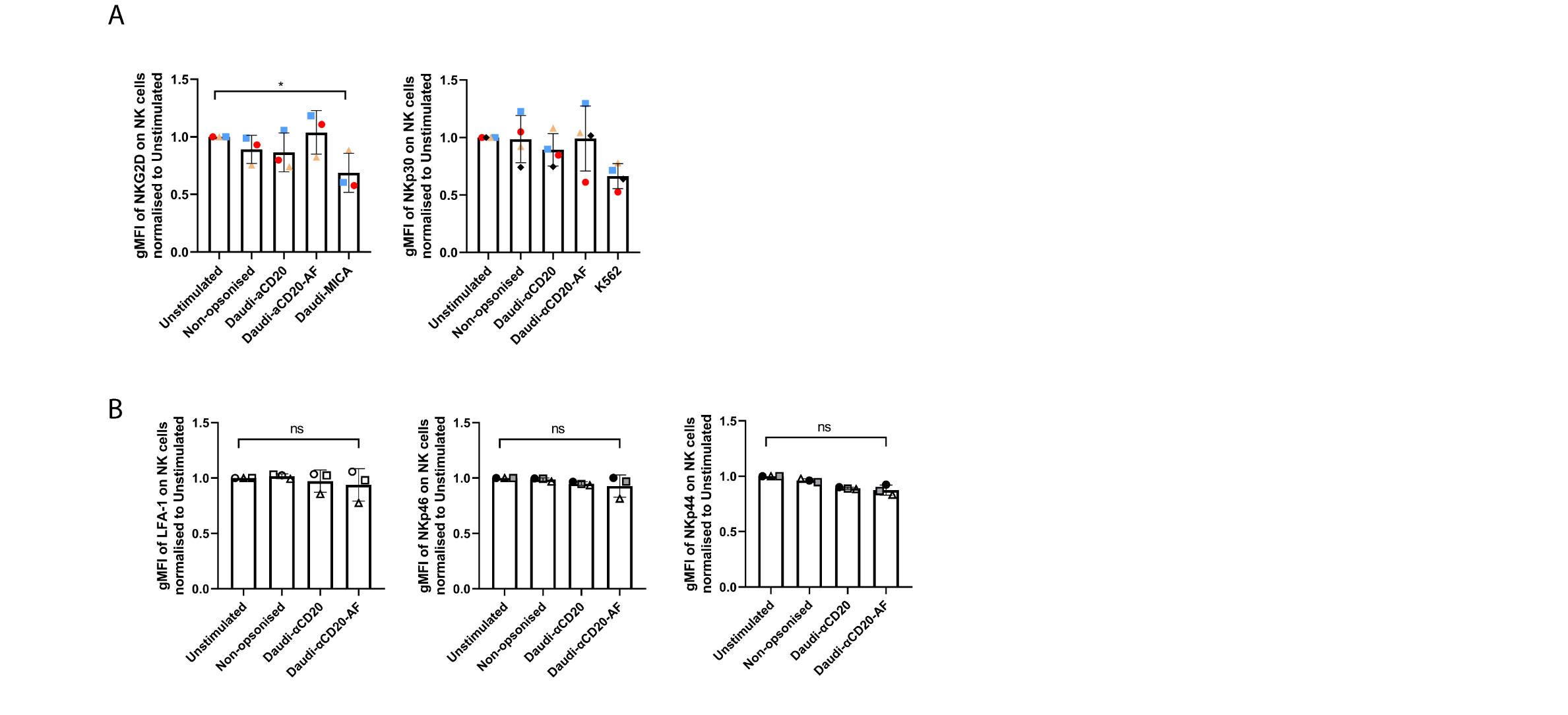
**

**Supplementary Figure 2. Expression of surface markers located in the immune synapse of primary NK cells stimulated by different conditions. (A-B)** Primary NK cells were co-incubated with variably opsonized 221 target cells in a E:T ratio = 1:3 for 3 hours and protein surface expression was measured by flow cytometry. **(A)** Graphs show the gMFI of NKG2D or NKp30 on activated NK cells compared to unstimulated NK cells. In addition, NK cells were co-incubated with Daudi-MICA and K562 cells as positive control for NKG2D and NKp30, respectively. (n = 3 distinct donors; mean ± SD). **(B)** Graphs show the gMFI of LFA-1, NKp46 or NKp44 on NK cells that had been co-incubated with variably opsonized 221 target cells in a E:T ratio = 1:3 for 3 hours compared to unstimulated NK cells. (n = 3 distinct donors; mean ± SD). *, P < 0.05; calculated by one-way ANOVA.


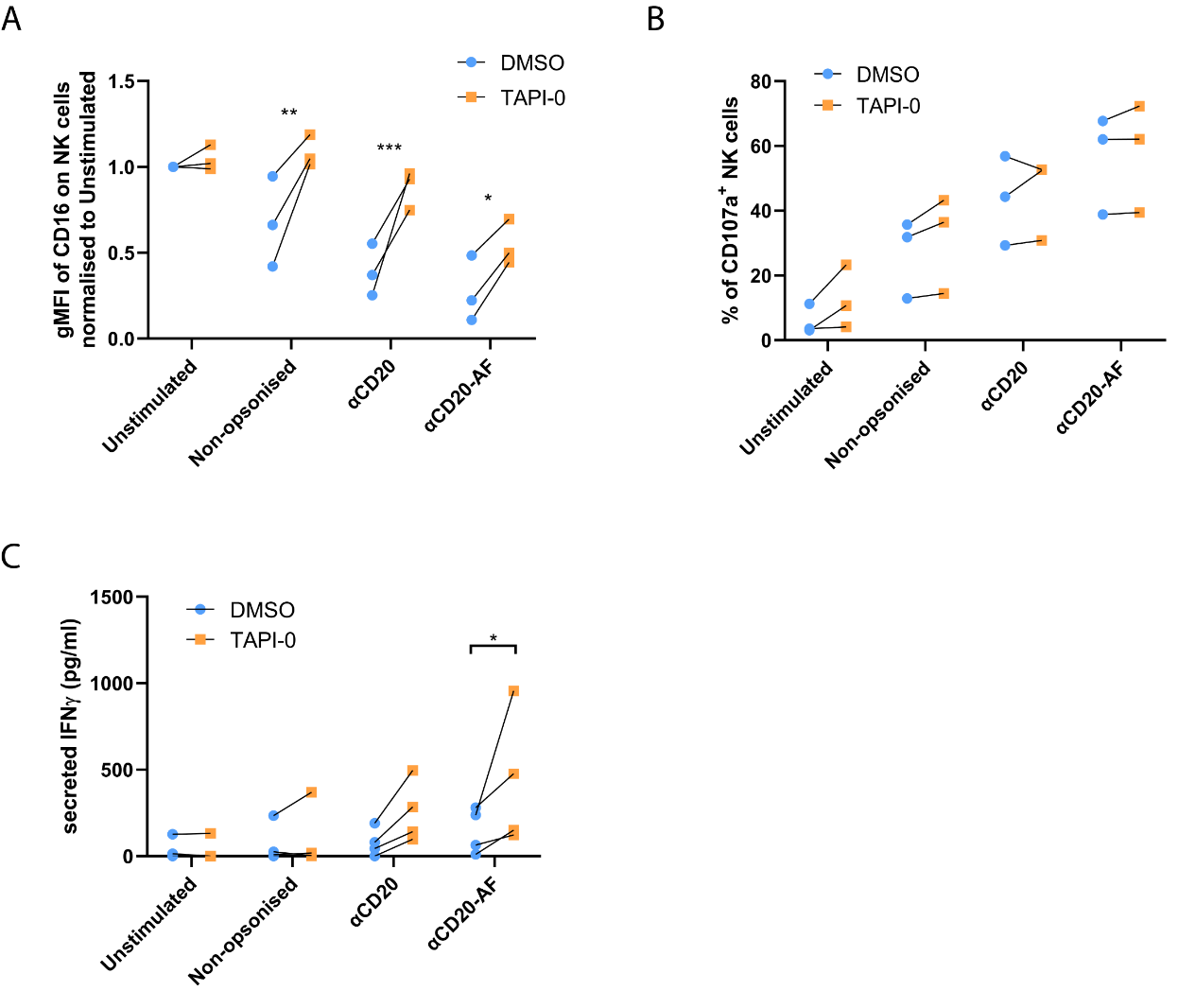


**Supplementary Figure 3. The effect of TAPI-0 in inhibiting CD16 shedding is not due to its vehicle, DMSO. (A-C)** NK cells were co-incubated with non-opsonised, anti-CD20 or anti-CD20-AF opsonised targets 221 target cells (E:T = 1:2) for 4 hours in the presence of the ADAM17 inhibitor, TAPI-0 (5 μM) or DMSO as a vehicle only control. Following co-incubation **(A)** surface CD16 expression and **(B)** the percentage of CD107a^+^, degranulating NK cells was assessed (mean ± SD, n=3 distinct donors). **(C)** NK cells were co-incubated with non-opsonised, anti-CD20 or anti-CD20-AF opsonised 221 target cells in the presence of DMSO or TAPI-0 inhibitor for 24 hours and the amount of IFNγ was measured (mean ± SD, n=4 distinct donors). *, P < 0.05; **, P < 0.01; ***, P < 0.001, calculated by multiple t-tests (A-C).

**
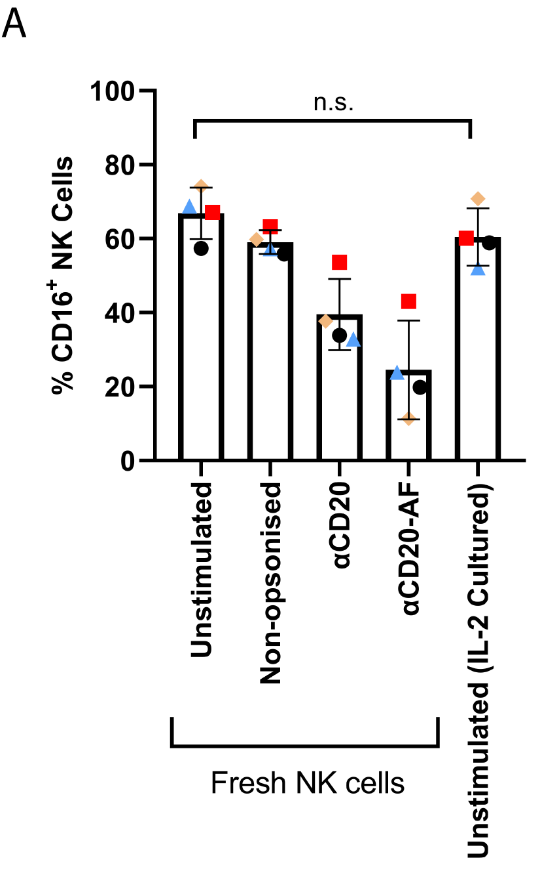
**

**Supplementary Figure 4. CD16 expression on freshly isolated primary NK cells compared with NK cells cultured in low-dose IL-2 for 6 days. (A)** Graph shows the percent of CD16^+^ NK cells on freshly isolated primary NK cells compared with IL-2 cultured NK cells without stimulation or for freshly isolated NK cells following a 2-hour co-incubation with non-opsonised, anti-CD20 or anti-CD20-AF opsonised 221 cells (mean ± SD, n = 4 distinct donors).


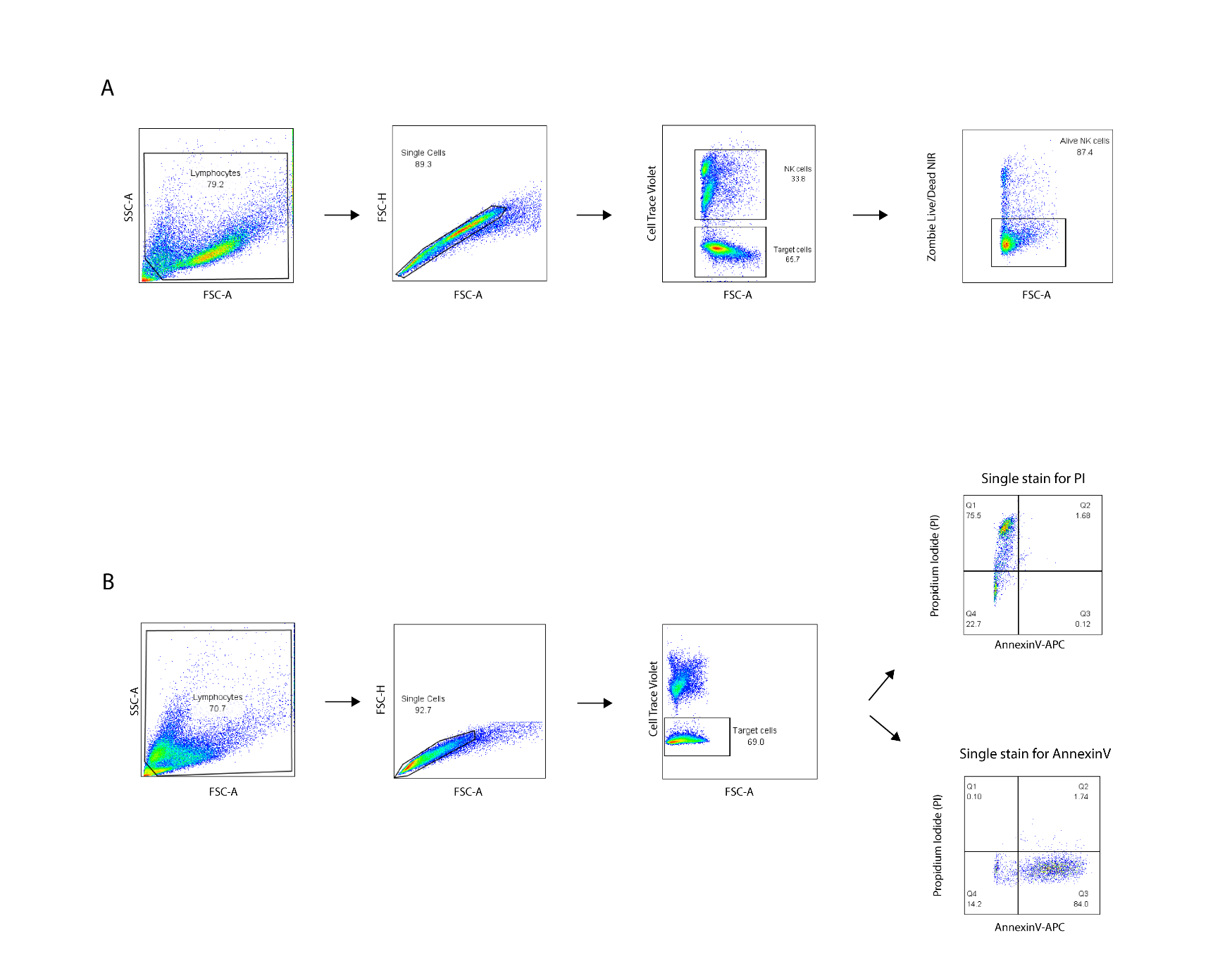


**Supplementary Figure 5. Gating strategy for Flow cytometric experiments.** **(A-B).** Co-cultures were firstly gated for Lymphocytes (FSC-A vs SSC-A) and then for single cells (FSC-H vs FSC-A). Next, co-cultured cells were divided based on Cell Trace Violet staining (NK cells vs Target cells). **(A)** In most experiments NK cells were then gated to identify live and dead cells based on staining with Zombie NIR, with live cells then assessed for further markers. **(B)** Alternatively, in killing assays, targets cells were analysed with gating based on single stain controls as shown at the bottom right (single stains for Propidium Iodide (PI) or Annexin V).

**Supplementary Videos Legends**

**Supplementary Video 1.** Opsonisation with anti-CD20-AF triggered quick lysis of two targets sequentially.

**Supplementary Video 2.** Opsonisation with anti-CD20 mAb triggered slow lysis of two targets sequentially.

**Supplementary Video 3.** Serial killing of two opsonised targets without prior detachment from the first target encountered.

**Supplementary Video 4.** Lysis of two anti-CD20-AF opsonised 221 target cells simultaneously

**Supplementary Video 5.** Serial killing of four anti-CD20-AF-opsonised 221 target cells.

**Supplementary Video 6.** Serial killing of five anti-CD20-AF-opsonised 221 target cells (3D Matrigel).

**Supplementary Video 7.** Lysis of three targets serially without prior detachment (3D Matrigel).

**Supplementary Video 8.** Lysis of four anti-CD20-AF-opsonised targets sequentially (3D Matrigel).

**Supplementary Video 9.** Lysis of multiple target cells simultaneously (3D Matrigel).

**Supplementary Video 10.** Slow lysis of anti-CD20-AF-opsonised target in the presence of TAPI-0 (no detachment).

**Supplementary Video 11.** Fast lysis of anti-CD20-opsonised target in the absence of TAPI-0 (detachment).

**Supplementary Video 12.** Multiple serial killing of opsonised target cells, boosted by TAPI-0
